# Angiogenesis during diabetic wound repair: from mechanism to therapy opportunity

**DOI:** 10.1093/burnst/tkae052

**Published:** 2025-02-07

**Authors:** Kang Huang, Bobin Mi, Yuan Xiong, Zicai Fu, Wenyun Zhou, Wanjun Liu, Guohui Liu, Guandong Dai

**Affiliations:** Department of Orthopedics, Southern Medical University Pingshan Hospital, No. 19 Renmin Street, Pingshan District, Shenzhen City, Guangdong Province, 518118, P.R. China; Department of Orthopedics, Pingshan District Peoples’Hospital of Shenzhen, No. 19 Renmin Street, Pingshan District, Shenzhen City, Guangdong Province, 518118, P.R. China; Department of Orthopedics, Southern Medical University Pingshan Hospital, No. 19 Renmin Street, Pingshan District, Shenzhen City, Guangdong Province, 518118, P.R. China; Department of Orthopedics, Pingshan District Peoples’Hospital of Shenzhen, No. 19 Renmin Street, Pingshan District, Shenzhen City, Guangdong Province, 518118, P.R. China; Department of Orthopedics, Southern Medical University Pingshan Hospital, No. 19 Renmin Street, Pingshan District, Shenzhen City, Guangdong Province, 518118, P.R. China; Department of Orthopedics, Pingshan District Peoples’Hospital of Shenzhen, No. 19 Renmin Street, Pingshan District, Shenzhen City, Guangdong Province, 518118, P.R. China; Department of Orthopedics, Southern Medical University Pingshan Hospital, No. 19 Renmin Street, Pingshan District, Shenzhen City, Guangdong Province, 518118, P.R. China; Department of Orthopedics, Pingshan District Peoples’Hospital of Shenzhen, No. 19 Renmin Street, Pingshan District, Shenzhen City, Guangdong Province, 518118, P.R. China; Department of Orthopedics, Southern Medical University Pingshan Hospital, No. 19 Renmin Street, Pingshan District, Shenzhen City, Guangdong Province, 518118, P.R. China; Department of Orthopedics, Pingshan District Peoples’Hospital of Shenzhen, No. 19 Renmin Street, Pingshan District, Shenzhen City, Guangdong Province, 518118, P.R. China; Department of Orthopedics, Southern Medical University Pingshan Hospital, No. 19 Renmin Street, Pingshan District, Shenzhen City, Guangdong Province, 518118, P.R. China; Department of Orthopedics, Pingshan District Peoples’Hospital of Shenzhen, No. 19 Renmin Street, Pingshan District, Shenzhen City, Guangdong Province, 518118, P.R. China; Department of Orthopedics, Southern Medical University Pingshan Hospital, No. 19 Renmin Street, Pingshan District, Shenzhen City, Guangdong Province, 518118, P.R. China; Department of Orthopedics, Pingshan District Peoples’Hospital of Shenzhen, No. 19 Renmin Street, Pingshan District, Shenzhen City, Guangdong Province, 518118, P.R. China; Department of Orthopedics, Southern Medical University Pingshan Hospital, No. 19 Renmin Street, Pingshan District, Shenzhen City, Guangdong Province, 518118, P.R. China; Department of Orthopedics, Pingshan District Peoples’Hospital of Shenzhen, No. 19 Renmin Street, Pingshan District, Shenzhen City, Guangdong Province, 518118, P.R. China

**Keywords:** Angiogenesis, Diabetes mellitus, Diabetic foot ulcers, Timing of treatment, Diabetic wound

## Abstract

Diabetes mellitus, a pervasive chronic metabolic disorder, is often associated with complications such as impaired wound healing. Various factors, most notably vascular deficiency, govern the wound repair process in diabetic patients, significantly impeding diabetic wound healing; therefore, angiogenesis and its role in diabetic wound repair have emerged as important areas of research. This review aims to delve into the mechanisms of angiogenesis, the effects of diabetes on angiogenesis, and the association between angiogenesis and diabetic wound repair. This will ultimately offer valuable guidance regarding the ideal timing of diabetic wound treatment in a clinical setting.

## Background

Recognized as a pressing global health concern, diabetes mellitus is a widespread and severe metabolic disorder. Characterized by microvascular complications, it is often associated with diabetic ulcers, a common complication. These ulcers are known for their delayed healing, high recurrence rate, risk of amputation, and elevated mortality, placing a significant psychological and economic strain on patients, their families, and society at large. It is noteworthy that the estimated number of cases of diabetes worldwide was 529 million in 2021, with projections suggesting a rise to 1.31 billion by 2050 [1]. Roughly 25% of diabetic patients are estimated to develop chronic nonhealing wounds [2]. Research shows a concerning trend, with the 1-year recurrence rate of diabetic foot ulcer repair of ~42%, and a 5-year recurrence rate nearing 65% [3]. Further studies indicate that most patients diagnosed with diabetic ulcers face amputation within 4 years, and the 5-year mortality rate is ~30% [3].

Current conventional treatment for diabetic wounds encompasses glycemic control, necrotic tissue debridement and infection management, dressing application, vascular reconstruction, and wound offloading. However, the overall effectiveness of these methods is unsatisfactory. This underscores the need for a more comprehensive understanding and approach. Wound repair is a dynamic and intricate process that is dependent on well-coordinated and precise interactions across coagulation, inflammation, proliferation, and remodeling phases, all of which converge to enhance wound healing. However, prolonged hyperglycemia disrupts the inflammatory response and impedes granulation tissue formation, angiogenesis, and epithelialization within the wound. More critically, the patient’s capacity for vascular regeneration is often severely hampered when diabetic ulcers present clinically. This suggests that successful diabetic wound repair extends beyond simply improving dressing changes or removing necrotic tissue. Targeted interventions at different stages of the wound repair process are vital in clinical practice. Therefore, examining the specific mechanisms of angiogenesis during diabetic wound repair is of significant clinical importance as it contributes to achieving a deeper understanding of the intricate processes involved and assists in the development of more appropriate treatment strategies and timelines.

In the present review, we focus on the mechanisms of angiogenesis in diabetic wound repair, bridging the gap between basic science and clinical practice and incorporating recent advancements in the field.

## Review

### Repercussions of diabetes on the process of wound healing

Wound healing is a multifaceted and dynamic process that aims at restoring skin structure and function. It unfolds through several overlapping stages: clotting, inflammation, cellular and matrix proliferation, and tissue remodeling. These phases involve the participation of various skin epithelial cells, fibroblasts, endothelial cells, recruited immune cells, and their associated extracellular matrix (ECM) [4]. Upon injury, a fibrin clot rapidly forms to halt blood loss and microbial invasion, initiating the clotting phase. This involves platelet aggregation and the release of fibrinogen fragments, growth factors, and proinflammatory mediators [5]. In the subsequent inflammatory phase, neutrophils and monocytes are recruited to clear debris and pathogens while releasing key growth factors and mediators that drive wound repair [6]. As inflammation subsides, the proliferative phase of wound healing, which is characterized by the formation of new tissue and blood vessels, begins as a synthesized matrix fills the injured area, restoring skin function and structural integrity. Finally, the remodeling phase strengthens the ECM, optimizes vascular structure, and achieves complete wound repair [7].

Wound healing is intricately regulated by repair cells, immune cells, cytokines, and signaling pathways. An imbalance in any of these elements can lead to delayed wound healing or nonhealing wounds, which is prevalent in conditions like diabetes, where chronic hyperglycemia impairs endothelial cell function, leading to vascular damage and ischemia, hindering wound healing [8]. Consequently, angiogenesis in diabetic wound repair is a key focus of research.

Unlike acute wounds that typically heal rapidly, wound repair in diabetic patients is often prolonged and may become chronic due to persistent high blood glucose levels that inhibit neutrophil function and cause peripheral vascular disease, tissue ischemia, and endothelial cell dysfunction [9,10]. This disrupts cell migration and proliferation, increasing the risk of infection and impeding healing [11]. Diabetic wounds exhibit persistent inflammatory responses, impaired tissue maturation, reduced tensile strength, and diminished angiogenesis [12,13]. Notably, impaired angiogenesis in diabetic patients manifests as reduced endothelial cell function, compromised blood vessel formation, and pericyte recruitment inhibition [14,15].

In conclusion, diabetes profoundly affects wound healing through various mechanisms, notably impaired angiogenesis. Thus, promoting angiogenesis could enhance diabetic wound repair. Future research should explore these effects and mechanisms further to develop more effective therapeutic strategies.

### Mechanism of angiogenesis during wound healing

Angiogenesis refers to the process by which new blood vessels differentiate from existing ones and grow toward the wound site. It plays a crucial role in tissue repair, as nourishment and oxygen delivery heavily rely on blood vessels within a 200 μm radius. Without proper blood supply, cellular survival is compromised [16]. The intricate process of angiogenesis in wound healing involves the interaction of various cell types and signaling molecules [17]. During the dynamic repair process, hematopoietic stem cells are guided to the wound site by various signaling molecules, where they are stimulated and activated by factors such as inducible cell donors and basic immune cells. This activation leads to the release of platelet chemotactic factors, tumor necrosis factor-alpha (TNF-α), and other factors, promoting endothelial cell formation and ECM generation. These molecules also recruit surrounding vascular cells, facilitating their proliferation and differentiation into endothelial cells, ultimately organizing them into a new vascular network. Angiogenesis, which is initiated after the recruitment of immune cells, primarily occurs during the wound healing phase, with the maturation of newly formed blood vessels and tissue remodeling as the ultimate goal [18]. Normally, angiogenesis involves the proliferation, migration, and lumen formation of endothelial cells, as well as the differentiation of vascular smooth muscle cells and pericyte recruitment. Vascular repair is regulated by various cytokines and signaling pathways, including vascular endothelial growth factor (VEGF), platelet-derived growth factor (PDGF), and the Notch pathway [19]. The primary processes of angiogenesis involve platelet activation and aggregation, followed by the release of both angiogenesis inhibitors to halt blood vessel formation and various proinflammatory cytokines to promote the inflammatory response [20]. Subsequently, macrophages, epithelial cells, and lymphocytes release several proangiogenic cytokines, facilitating the formation and reconstruction of new blood vessels at the site of injury, thereby promoting wound healing [21].

**Figure 1:**
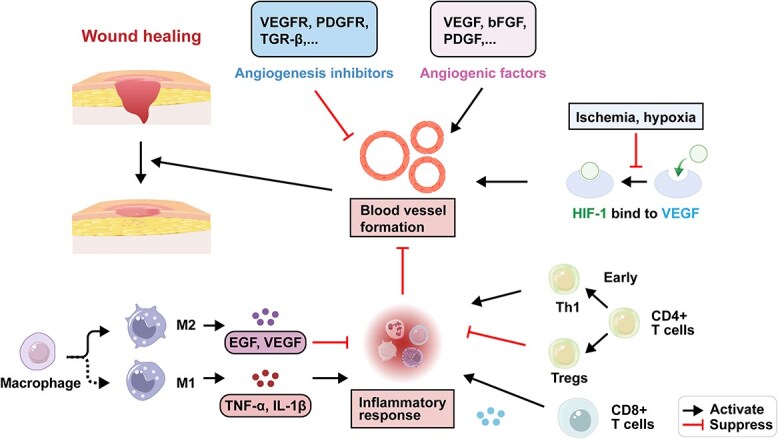
Mechanisms of angiogenesis during wound healing. *VEGFR* vascular endothelial growth factor receptors, *PDGFR* platelet-derived growth factor receptors. Figure created with BioRender.com

Once the wound is completely covered with regenerated epithelium, the healing process requires tissue remodeling and reconstruction. During the remodeling phase, fibroblasts differentiate into myofibroblasts and secrete a substantial amount of collagen molecules, which undergo covalent cross-linking to enhance the tensile strength of the wound. However, proangiogenic protein expression decreases in the newly formed blood vessels at the wound site, and the contraction and compression of collagen lead to a decrease in wound perfusion. As a result, the mechanical stress on the blood vessel walls surrounding the wound decreases, ultimately leading to microvascular occlusion and degeneration [22]. Moreover, granulation tissue matures and transforms into scar tissue containing a few blood vessels, with the balance of collagen fiber synthesis and degradation mediated by matrix metalloproteinases (MMPs) and myofibroblasts [23]. Therefore, wound angiogenesis is accomplished within the dynamic equilibrium of angiogenesis inhibitors and proangiogenic factors, which is significantly disrupted by diabetes, hindering the smooth progression of this dynamic process (Fig. 1).

### Factors influencing angiogenesis

Angiogenesis, a critical process in wound healing, is intricately regulated by both promoting and inhibiting factors. These factors influence endothelial cell activities such as proliferation, migration, and tubule formation, thereby affecting the formation and growth of new blood vessels. Common angiogenesis inhibitors include inhibitors of VEGF receptors, PDGF receptors, transforming growth factor-beta (TGF-β), angiostatin, endostatin, thrombospondin-1, and others [24,25]. In contrast, the process is promoted by angiogenic factors like VEGF, basic fibroblast growth factor (bFGF), PDGF, and others [26].

MMPs are a class of soluble proteases that can degrade the ECM. They not only facilitate matrix degradation but also enhance the effects of various angiogenic factors, thus promoting angiogenesis. bFGF stimulates the synthesis of MMPs and urokinase-type plasminogen activator, leading to basal membrane degradation and migration to the site of injury, thus inducing mature blood vessel formation. It is noteworthy that in patients with diabetic foot, there is an observed increase in FGF-2 expression but a decrease in VEGF expression [27]. Interestingly, this pattern is reversed in patients with uncomplicated diabetes. These observations suggest that the expression of FGF-2 and VEGF presents unique temporal patterns, and their interplay plays a vital role in the delayed healing of diabetic foot ulcers.

### Role of hypoxia and hypoxia-inducible factors

One of the main reasons for delayed healing is the peripheral circulation compromise, which leads to severe ischemia and hypoxia in the local wound area due to high blood- glucose levels. This is further exacerbated by impaired collateral vessel formation, which contributes to the establishment of a hypoxic microenvironment. Under such conditions, cells produce transcriptional activation factors known as hypoxia-inducible factors (HIFs). HIFs include HIF-1α, HIF-2α, HIF-3α, and HIF-1β/ARNT hydrocarbon receptor nuclear translocator also known as HIF-1β.

HIF-1β expression in the cell nucleus is independent of oxygen concentration, thereby ensuring the structural stability of HIF [28]. However, both HIF-1α and HIF-2α subunits are sensitive to oxygen levels, exhibiting high expression under hypoxic conditions [29,30].

Several studies have highlighted the fact that during the wound healing process, tissue hypoxia can lead to vascular rupture, increased oxygen consumption, and an inflammatory response. However, in a hyperglycemic environment, such as the one created by diabetic wound ulceration, ischemia and hypoxia-induced HIF do not sufficiently compensate for the damage of the high-sugar environment. This impairment leads to a decrease in the expression levels of downstream factors like VEGF that are crucial for angiogenesis. Consequently, tissue hypoxia is further exacerbated, leading to circulatory disorders and ultimately impaired wound healing [31].

### Impact of the local inflammatory response and immune microenvironment

There is a close relationship between local inflammation and angiogenesis in tissue regeneration. Inflammation promotes neovascularization, while the newly formed blood vessels provide the oxygen and nutrients necessary for the inflammatory response.

The immune microenvironment formed by local inflammatory reactions plays a crucial role in tissue regeneration. Tissue regeneration typically begins with an early immune-inflammatory response, which triggers immune cell enhancement and inflammatory cytokine and chemokine secretion, subsequently mobilizing and recruiting immune cells to the site of injury. Simultaneously, stem cells can respond to the immune microenvironment during the tissue regeneration process and regulate the immune-inflammatory response [32]. These inflammatory factors attract and activate surrounding vascular cells, thereby stimulating endothelial cell proliferation and migration. As the building blocks of newly formed blood vessels, they begin to generate supportive cells in close proximity, secreting matrix molecules to repair the ECM region, ultimately leading to the formation of a new blood vessel.

However, the presence of hyperglycemia in diabetes significantly affects wound healing and tissue repair. Local inflammation and angiogenesis are two crucial processes in wound repair. The specific relationship between the inflammatory response and angiogenesis mainly includes the dynamic balance of M1/M2 macrophages and the balance of CD4^+^/CD8^+^ T cells.

Macrophages, which are vital components of both innate and adaptive immunity, regulate the inflammatory response. They differentiate from monocytes during the latter stages of inflammation, functioning not only to clear senescent neutrophils and tissue debris in the wound site but also to engulf pathogens and present antigens to T cells, mediating the progression of inflammation. Recent studies have demonstrated that macrophages can switch phenotypically from the proinflammatory M1 phenotype to the healing-associated M2 phenotype. Macrophages secrete both proinflammatory factors, such as TNF-α and interleukin (IL)-1β, and anti-inflammatory factors like TGF-β1, and growth factors including EGF and VEGF [33,34]. These anti-inflammatory factors and growth factors crucially regulate vascular responses, promoting angiogenesis and re-epithelialization. They also stimulate the migration, proliferation, and differentiation of fibroblasts and wound repair cells, fostering collagen and ECM synthesis [35].

Proinflammatory M1 macrophages exhibit robust phagocytic abilities and actively partake in the inflammatory response by eradicating pathogens and debris within local tissues [36]. As a result, M1 macrophages secrete proinflammatory cytokines and chemokines, such as IL-1β, IL-6, IL-12, IL-23, and chemokine (C-X-C motif) ligands [37]. Conversely, M2 macrophages, characterized by anti-inflammatory properties, play an essential role in regulating processes such as angiogenesis, fibroblast regeneration, myofibroblast differentiation, and collagen synthesis [38]. Certain cytokines, including the granulocyte–macrophage colony-stimulating factor, induce macrophage polarization toward the M2 phenotype. This polarization results in the secretion of numerous anti-inflammatory molecules, enhancing anti-inflammatory activity and promoting tissue regeneration [39].

As critical immune cells, T cells are generally classified into two major subsets: CD4+ T cells and CD8+ T cells. Among them, regulatory T cells (Tregs) are fundamental in preserving immune tolerance within the body. They actively inhibit the activity of potentially self-reactive T cells, thereby controlling the immune system and preventing the development of autoimmune diseases [40]. Tregs, CD4+ cells, and CD8+ cells are vital subtypes of immune cells in the human immune system, and they play key roles in the maintenance of immune system equilibrium and functionality. CD4+ cells are particularly important in directing the body’s defense against pathogens and facilitating the activation and proliferation of other immune cells [41]. CD8+ T cells can be further activated into effector T cells, known as cytotoxic T lymphocytes. They are primarily located in the tonsils, spleen, and other organs, with their main function being the elimination of pathogens and abnormal cells within the body [42]. During the wound healing process, CD4+ and CD8+ T cells are summoned to the wound site, peaking around 5–10 and 7–10 days postinjury, respectively. These two types of T cells have distinct roles in mediating wound regeneration: CD4+ T cells are associated with enhanced repair, while CD8+ T cells are implicated in the healing of damaged tissue [43].

CD4+ cells can foster the early inflammatory response during wound healing. They can secrete various cytokines such as TNF-α, activating the inflammatory response and aiding in the clearance of pathogens and necrotic tissue at the wound site. Additionally, they can differentiate into different subsets, including Th1, Th2, Th17, and Treg cells, each with unique functions in wound healing. For example, Th1 cells can boost the inflammatory response and fibrous tissue formation, while Tregs can suppress the inflammatory response and promote wound healing. Therefore, balanced regulation of CD4+ cells ensures immune equilibrium during wound healing, avoiding excessive inflammation or immune suppression [44]. CD8+ cells play a central role in promoting wound clearance and repair by eliminating infected cells and eradicating pathogens. Additionally, CD8+ cells secrete various cytokines to enhance the inflammatory response and facilitate fibrous tissue formation during wound healing [45].

The balance between CD4+ and CD8+ T cells plays a critical role in wound healing. By regulating the differentiation and function of CD4+ cells, the immune balance and modulation of inflammatory responses can be achieved during wound repair. Simultaneously, the involvement of CD8+ cells helps promote wound clearance and repair. Therefore, maintaining equilibrium between CD4+ and CD8+ cells is vital for successful wound healing outcomes.

### Key role of vascular stem cells

Vascular stem cells, also known as endothelial progenitor cells (EPCs), play a crucial role in angiogenesis and subsequent wound healing. These cells, which are derived from the bone marrow, can differentiate into mature endothelial cells, contributing to the formation of new blood vessels [46]. EPCs are mobilized from the bone marrow into the peripheral circulation in response to various stimuli, including ischemia, tissue injury, and the presence of specific growth factors. Once in circulation, these cells migrate to sites of vascular injury or ischemia, where they participate in neovascularization. The recruitment and incorporation of EPCs into the newly formed vasculature involve several steps. First, EPCs are mobilized from the bone marrow and enter the circulation. Next, they are recruited to the site of injury or ischemia through a process mediated by various chemokines and adhesion molecules. Upon reaching the target site, EPCs proliferate, differentiate, and incorporate the existing vasculature, contributing to angiogenesis [47]. Several factors, including VEGF, stromal cell-derived factor-1, and nitric oxide (NO), play crucial roles in the mobilization, recruitment, and differentiation of EPCs [48].

In the context of wound healing, EPCs contribute to angiogenesis, which is essential for the delivery of oxygen and nutrients to the injured tissue, as well as waste removal. The presence of functional EPCs has been associated with improved wound healing outcomes, while impairments in EPC function or number have been associated with delayed or impaired wound healing [49]. In diabetes, the number and function of EPCs are often compromised, leading to impaired angiogenesis and delayed wound healing. This dysfunction is attributed to various factors, including hyperglycemia, oxidative stress, and chronic inflammation, which can affect the mobilization, survival, and function of EPCs [50]. Strategies aimed at enhancing the number and function of EPCs, such as cell therapy or specific signaling pathway modulation, have been explored as potential therapeutic approaches for improving wound healing in diabetic patients [51]. Overall, vascular stem cells play a crucial role in angiogenesis and subsequent wound healing, and their dysfunction contributes to the wound healing impairment observed in diabetes.

### Relationship between diabetes, diabetic wounds, and angiogenesis

Diabetic wounds, a common complication of diabetes, often exhibit delayed healing, mainly due to abnormal angiogenesis in the diabetic wound bed [52]. Studies have found that the vascular endothelial cells in the ulcer tissue of patients with diabetic wounds are disordered and unable to effectively form a complete lumen-like structure [53]. Therefore, promoting angiogenesis could be key to improving diabetic wound healing. However, in diabetics, prolonged hyperglycemia, hyperlipidemia, and oxidative stress can disrupt various metabolic pathways during wound healing, leading to cellular dysregulation and impaired reparative cell functionality. These factors contribute to the reduced neovascularization seen in diabetic wounds [54]. In this environment, the local inflammatory process is intensified, leading to the excessive production of chemotactic factors, proteases, and other substances that hinder the proper release of essential growth factors and vascular endothelial cells, causing secondary damage to the overall healing process. Furthermore, when angiogenesis is abnormal or excessive, these newly formed blood vessels tend to be fragile and prone to leakage and rupture, thereby exacerbating inflammation and worsening the wound’s condition.

Under the influence of these various factors, angiogenesis in diabetic wounds is continuously diminished. This not only leads to inadequate oxygen and nutrient supply to the wound site but also results in a reduced influx of reparative cells through neovascularization, thus delaying wound healing. Current research suggests that the decrease in neovascular density in diabetic ulcer patients is primarily associated with endothelial cell dysfunction, growth factor/inhibitor imbalance, MMP/tissue inhibitor of metalloproteinase (TIMP) equilibrium dysregulation, and aberrant inflammatory signaling pathways [55,56].

**Figure 2:**
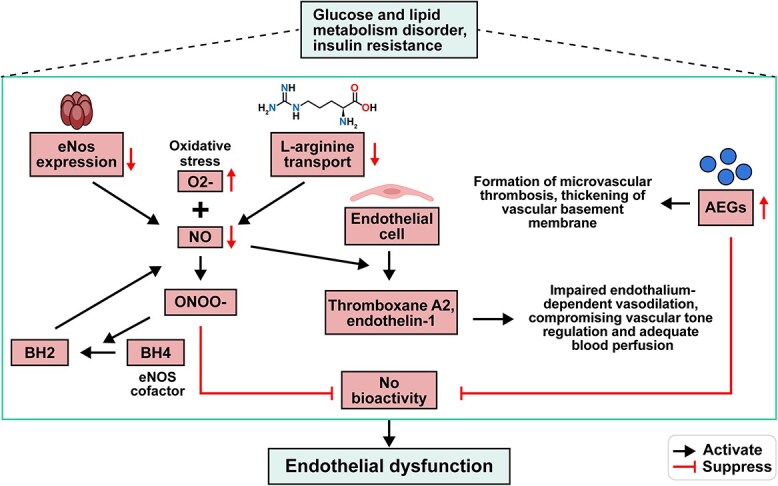
Endothelial cellular dysfunction. *O_2_*^*−*^ Superoxide anion, *ONOO*^*−*^ peroxynitrite, *BH2* dihydrobiopterin, *BH4* tetrahydrobiopterin. Figure created with BioRender.com

#### Endothelial cellular dysfunction in diabetic wounds

Endothelial cell functionality serves as the initiating factor and crucial link in promoting neovascularization. Research has confirmed that in patients with type 2 diabetes, high blood-glucose, lipid metabolism disorders, insulin resistance, and oxidative stress can reduce the proliferation and migration ability of endothelial cells, inducing endothelial cell apoptosis [57,58]. Disturbances in glucose and lipid metabolism, as well as insulin resistance, can downregulate the expression of endothelial NO synthase (eNOS) and the transport capacity of endothelial cell membranes for L-arginine, leading to a decrease in the production of NO by eNOS. Under conditions of oxidative stress in the wound, excessive superoxide anions (O_2_^−^) can rapidly combine with NO to generate peroxynitrite (ONOO^−^), a potent oxidizing agent. This not only reduces the bioactivity of NO but also oxidizes the crucial cofactor tetrahydrobiopterin (BH4) of eNOS into dihydrobiopterin (BH2), causing eNOS uncoupling and an increase in O_2_^−^ production, ultimately leading to endothelial dysfunction [59]. Moreover, the high rate of generation of advanced glycation end products (AGEs) in diabetic wounds not only inactivates NO and increases vascular permeability but also impairs vascular dilation, exacerbating endothelial dysfunction [60]. The reduced NO activity and endothelial cell damage caused by AGEs in the wound promote the expression of endothelial cell adhesion molecules and the adhesion and aggregation of platelets, thereby increasing endothelial cell permeability and facilitating leukocyte and platelet adhesion and aggregation. This leads to the formation of microvascular thrombosis and thickening of the vascular basement membrane [61]. The decreased expression of NO can also induce the secretion of vasoconstrictive substances such as thromboxane A2 and endothelin-1 by endothelial cells, leading to impaired endothelium-dependent vasodilation and compromising vascular tone regulation and adequate blood perfusion [62].

Upon the occurrence of a wound, diabetic patients experience an imbalance in the inflammatory response due to the paucity and compromised health status of endothelial cells, as well as the corresponding supporting stations and coordinating tissues of the associated blood vessels. This instability in overall vascular cell function leads to decreased dependence on microcirculation, predisposing patients to local microvascular inflammation (Fig. 2).

**Figure 3:**
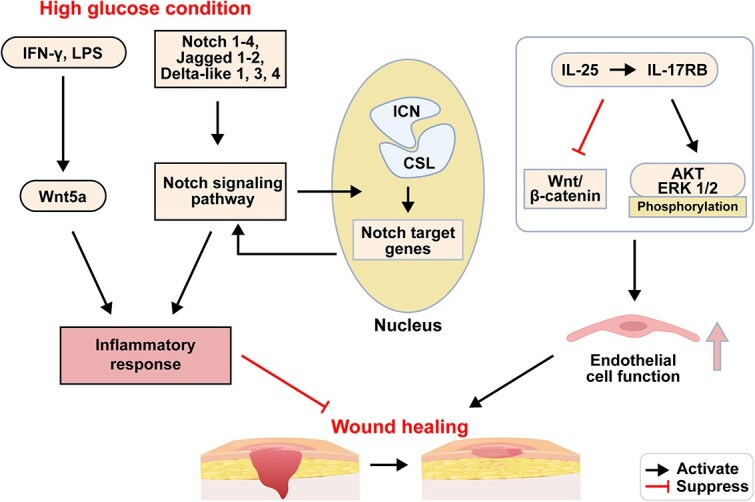
Inflammatory signaling pathways. IFN-γ interferon-gamma, LPS lipopolysaccharide. Figure created with BioRender.com

**Figure 4:**
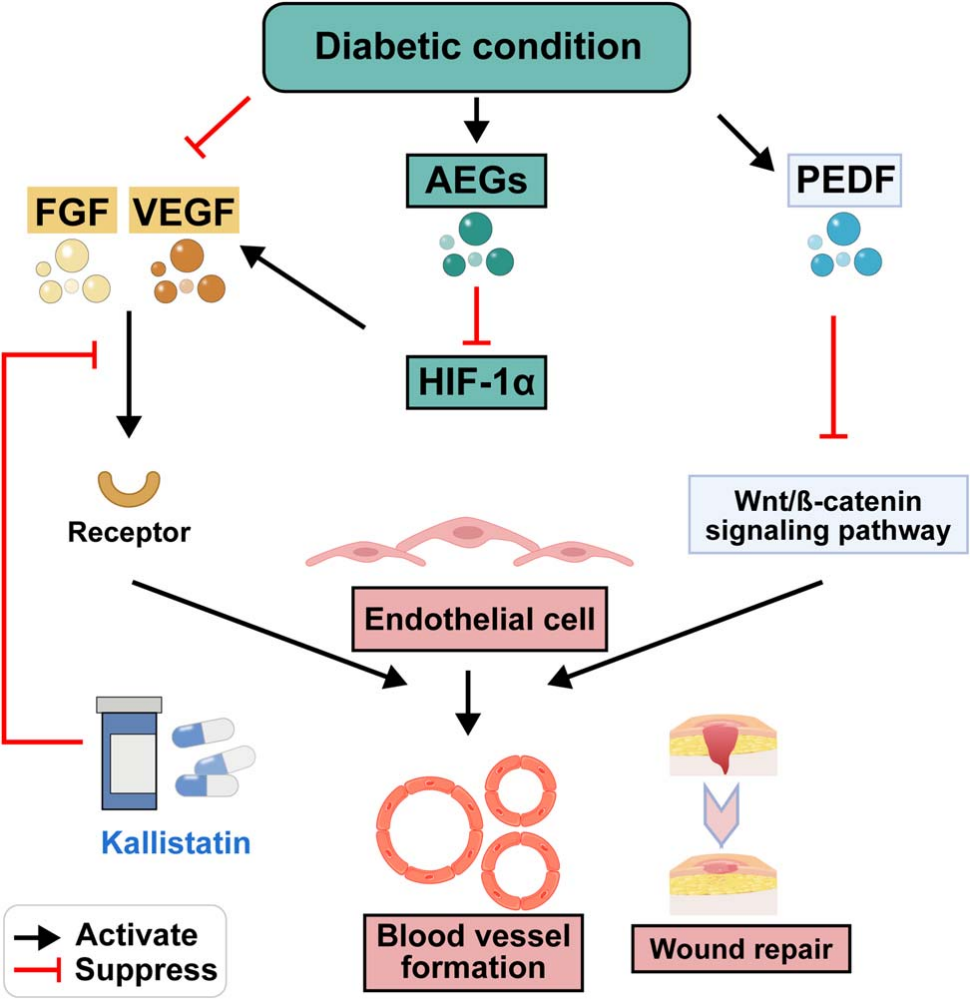
Balance of angiogenesis promoting factor/angiogenesis inhibiting factor. Figure created with BioRender.com

**Figure 5:**
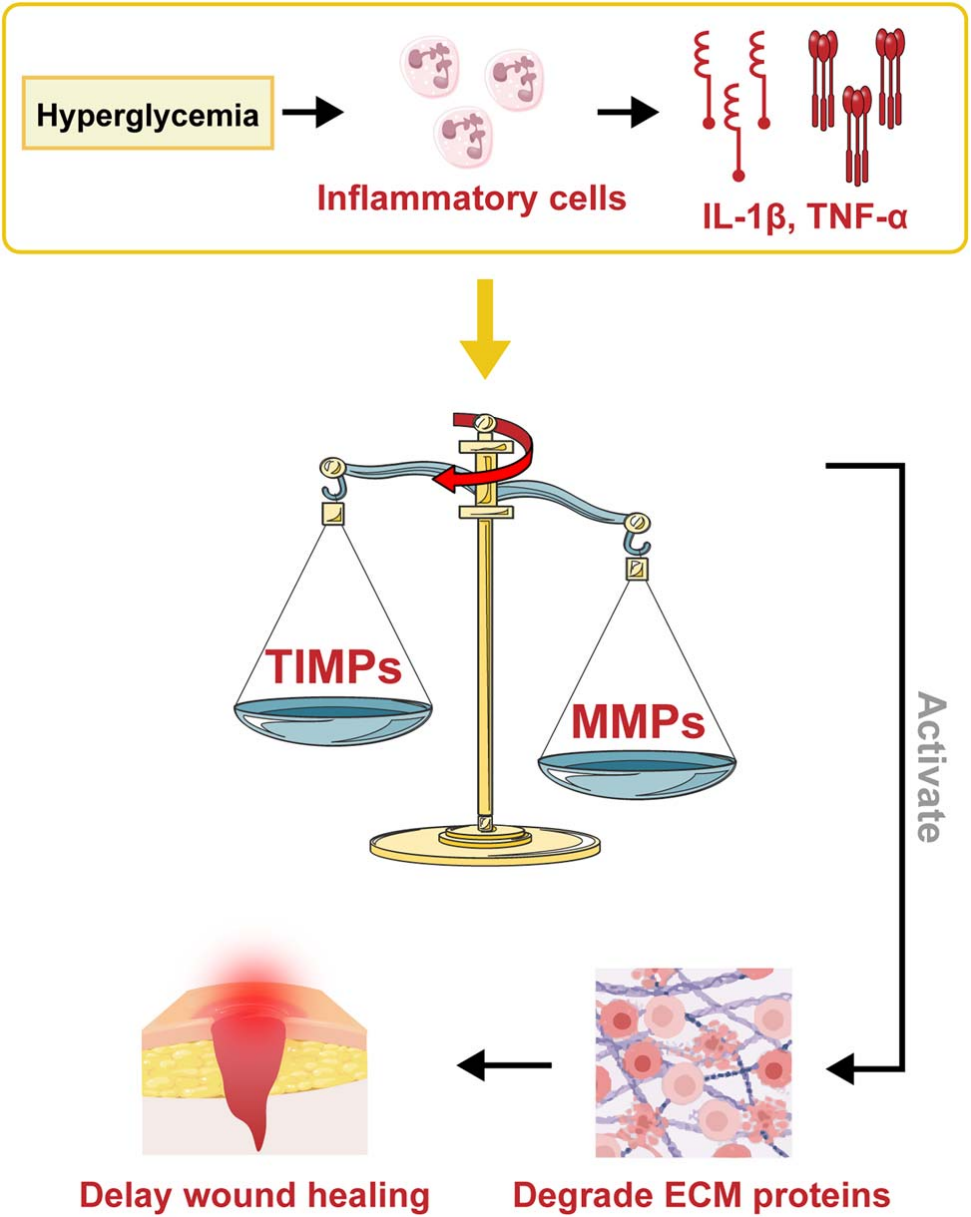
Balance of MMPs/TIMPs. Figure created with BioRender.com

#### Dysregulation of inflammatory signaling pathways

Diabetic wound healing impairment may be closely associated with a reduction in the expression of negative regulators of inflammation, leading to uncontrolled intracellular inflammatory pathway activation. This decrease in expression could also contribute to the persistent inflammatory state often observed in these cases [63]. Inflammation, as a crucial protective response, plays a key role in tissue regeneration and the elimination of triggering factors in damaged tissues. However, a detrimental inflammatory response can increase harmful triggers, particularly bacterial damage to tissues. Chronic unresolved inflammation can lead to pathological changes, manifested by the persistent nonhealing of diabetic wounds. At the cellular and molecular levels, traditional inflammatory cytokines and Wnt factors regulate tissue repair and regeneration in mammals [64]. Interferon-gamma and lipopolysaccharide effectively stimulate inflammatory factors, leading to significant Wnt5a upregulation and an ensuing improvement in the wound inflammatory response. The upregulation of IL-17 receptor B (IL-17RB) mediated by interleukin-25 improves endothelial cell function. IL-25-mediated IL-17RB signaling downregulates the Wnt/β-catenin pathway and induces the phosphorylation of AKT and extracellular regulated protein kinases 1/2 (ERK 1/2) under high-glucose conditions. The local administration of recombinant IL-25 protein improves vascular regeneration and collagen deposition in the wound bed, thereby ameliorating wound healing delays in patients with diabetes.

**Table 1:** Some of the drugs associated with promoting angiogenesis in diabetic wound therapy

**Drug/agent**	**Class**	**Mechanism of action**	**FDA approval status**
VEGF [77]	Growth factor	Stimulates angiogenesis by promoting endothelial cell proliferation, migration, and survival.	Not approved for diabetic wound healing.
PDGF [78]	Growth factor	Promotes angiogenesis and recruits cells involved in wound repair.	Approved for diabetic foot ulcers (Regranex).
Gene therapy (e.g. VEGF, FGF) [79]	Gene delivery	Delivery of genes encoding pro-angiogenic factors to stimulate new blood vessel formation.	Not approved for diabetic wound healing.
miRNAs (e.g. miR-126, miR-210) [80,81]	microRNAs	Regulate the expression of pro-angiogenic or anti-angiogenic genes at the post-transcriptional level.	Not approved for diabetic wound healing.
Angiotensin-converting enzyme (ACE) inhibitors [82]	Antihypertensive	Inhibit the breakdown of bradykinin, a pro-angiogenic factor.	Approved for hypertension, not specifically for diabetic wound healing.
Statins [83]	Cholesterol-lowering	Upregulate eNOS and promote angiogenesis.	Approved for hyperlipidemia, not specifically for diabetic wound healing.
Antioxidants (e.g. vitamin C, vitamin E) [84,85]	Antioxidants	Scavenge reactive oxygen species and improve endothelial function, indirectly promoting angiogenesis.	Approved as dietary supplements, not specifically for diabetic wound healing.
HIF stabilizers [86]	Transcription factor modulators	Stabilize HIF, a transcription factor that regulates the expression of pro-angiogenic genes.	Not approved for diabetic wound healing.
Radix astragali (Huang Qi); radix angelicae sinensis (Dang Gui); Rhizoma chuanxiong (Chuan Xiong) [87]	Traditional Chinese medicine	Promotes angiogenesis and wound healing through various mechanisms, including antioxidant activity and modulation of growth factors.	Not approved by FDA for diabetics.

The Wnt/β-catenin signaling pathway, which is highly conserved throughout evolution, is a crucial regulatory process in wound healing. It primarily participates in biological processes such as cellular proliferation, apoptosis, and differentiation, playing a vital role in the promotion of wound angiogenesis, epithelial remodeling, and timely wound closure [65]. Additionally, due to the unstable blood glucose levels commonly observed in diabetic patients, chronic hyperglycemia has been found to not only acutely stimulate the insulin gene promoter but also lead to the loss of glucose responsiveness in the insulin-related gene promoter expression.

The Notch signaling pathway is of paramount importance in the early inflammatory stage of wound healing, guiding the production of macrophage-dependent inflammatory mediators. The canonical Notch signaling is crucial in directing the functionality of macrophages in wound repair and identifying transformative targets for diabetic wound treatment [66]. Notch signaling is activated upon interaction between membrane-bound Notch receptors and ligands (Jagged 1–2 and Delta-like 1, 3, 4). Upon activation, the Notch signaling pathway releases the active form, Intracellular Domain of Notch (ICN), which translocates to the nucleus and forms a complex with CSL proteins, facilitating the transcription of Notch target genes. Notch signaling is activated by hyperglycemic signals in diabetic skin, inducing the formation of a specific positive feedback loop involving the Delta-like 4–Notch1feedback loop [67] (Fig. 3).

**Table 2:** Clinical trials of promoting angiogenesis during diabetic wound treatment

**Clinical trial title**	**Treatment method**	**Key findings**	**Clinical trial number**	**Phase**
Translational development of ABCB5+ dermal mesenchymal stem cells for therapeutic induction of angiogenesis in non-healing diabetic foot ulcers [90].	Topical application of GMP-manufactured ABCB5+ mesenchymal stem cells	Significant reduction in wound surface area by median of 59–67% at week 12. No treatment-related adverse events observed.	NCT03267784	Phase I/IIa
Wound healing in diabetic foot ulcer patients using combined use of platelet-rich fibrin and hyaluronic acid, platelet-rich fibrin and placebo: an open label, randomized controlled trial [91].	Topical application of autologous platelet-rich fibrin (A-PRF) + hyaluronic acid (HA), A-PRF, or sodium chloride 0.9% (control)	Significant increase in VEGF levels and decrease in IL-6 levels with A-PRF + HA treatment. PRF + HA combination increased angiogenesis and reduced inflammation.	ID 0855/UN2.F1/ETIK/2018	Phase I
A multicenter clinical trial evaluating the durability of diabetic foot ulcer healing in ulcers treated with topical oxygen and standard of care *vs* standard of care alone 1 year post healing [92].	Topical oxygen therapy (TOT) in addition to standard of care *vs* standard of care alone	85% of TOT patients remained healed at 1 year compared to 60% in standard care group. Trend towards more durable closure with TOT treatment.	NOW.T-001	Phase I
Prospective, randomized, and controlled study of a human umbilical cord mesenchymal stem cell injection for treating diabetic foot ulcers [93].	Injection of human umbilical cord mesenchymal stem cells	Study ongoing to evaluate efficacy of stem cell injection for diabetic foot ulcers.	XYFY2021-KL124–02	Phase I
Stem cell mobilization with plerixafor and healing of diabetic ischemic wounds: a phase IIa, randomized, double-blind, placebo-controlled trial [94].	Injection of plerixafor (CXCR4 antagonist) or saline (placebo) in addition to standard therapy	No improvement in wound healing with plerixafor treatment compared to placebo. Potential adverse effect of mobilizing diabetic hematopoietic stem/progenitor cells on wound healing.	NCT02790957	Phase IIa
Efficacy of LL-37 cream in enhancing healing of diabetic foot ulcer: a randomized double-blind controlled trial [95].	Topical application of LL-37 cream	LL-37 cream enhanced healing rate of diabetic foot ulcer (DFU) with mild infection. No significant decrease in IL-1α and TNF-α levels or aerobic bacteria colonization.	NCT04098562	Phase 2
Improved healing of chronic diabetic foot wounds in a prospective randomized controlled multi-centre clinical trial with a microvascular tissue allograft [96].	Application of processed microvascular tissue (PMVT) allograft	Increased complete wound closure at 12 weeks, greater percent wound area reduction, decreased time to healing, and improved local neuropathy with PMVT treatment.	ISRCTN #24783859, Western IRB study #1175398 protocol #20171089, and South Shore reference #17–013	Phase I
Hyperbaric oxygen therapy in management of diabetic foot ulcers: indocyanine green angiography may be used as a biomarker to analyze perfusion and predict response to treatment [97].	Hyperbaric oxygen therapy (HBOT)	Improved perfusion in chronic wounds with HBOT. Indocyanine green angiography patterns may predict response to HBOT.	Not applicable	Not applicable
Treatment of chronic diabetic foot ulcers with adipose-derived stromal vascular fraction cell injections: safety and evidence of efficacy at 1 year [98].	Injection of autologous adipose-derived stromal vascular fraction (SVF) cells	Safe treatment with evidence of efficacy in wound healing and vascular repair/angiogenesis.	Not applicable	Phase I
Complete wound closure following a single topical application of a novel autologous homologous skin construct: first evaluation in an open-label, single-arm feasibility study in diabetic foot ulcers [99].	Topical application of autologous homologous skin construct (AHSC)	Successful closure of DFUs with a single application of AHSC in most cases. No adverse events related to AHSC treatment observed.	NCT03881254	Phase I
A prospective randomized controlled study of antibiotic bone cement in the treatment of diabetic foot ulcer [100].	Treatment with antibiotic bone cement *vs* silver sulfadiazine cream	Antibiotic bone cement promoted accelerated wound healing and improved local blood flow compared to silver sulfadiazine cream.	KY20212159-F-1	Phase I

#### Imbalance of angiogenesis-regulating factors

In normal physiological conditions, a delicate equilibrium exists between proangiogenic factors and angiogenic inhibitors, thereby ensuring proper vascular development and homeostasis. However, in certain disease states such as diabetes, this delicate balance is disrupted. For instance, VEGF, a chemotactic and stimulatory factor for endothelial cells, works in tandem with FGF to stimulate endothelial cell growth and facilitate wound angiogenesis. In patients with diabetic foot complications, VEGF expression is significantly lower than it is in diabetic patients without such complications, possibly due to impaired endothelial cell function, resulting in insufficient VEGF expression and subsequent hindrance of blood vessel formation, leading to nonhealing ulcers [68,69].

Pigment epithelium-derived factor (PEDF) is one of the most widely expressed angiogenic inhibitors in vascular endothelial cells and surrounding cells. It inhibits endothelial cell proliferation in a dose-dependent manner. Therefore, PEDF, which is recognized as the most promising endogenous anti-angiogenic factor, is associated with microvascular complications in diabetic patients [70]. Research has demonstrated that in individuals with high blood glucose levels, there is a heightened expression of PEDF in wound sites. Furthermore, it has been observed that PEDF can effectively inhibit endothelial progenitor cell activation and inhibition by suppressing the Wnt-ß catenin signaling pathway. This action impedes vascular formation at the site of diabetic mouse wounds, thereby hindering wound healing [71]. Kallistatin is another typical angiogenic inhibitor. Composed of two structural domains—the activation domain and the heparin-binding domain—kallistatin, through its heparin-binding domain, competitively inhibits the binding of various cytokines such as VEGF, TGF -β, and TNF-α to their respective receptors. This mechanism allows kallistatin to exert its anti-angiogenic and anti-inflammatory effects [72].

Studies have revealed that the gradual accumulation of AGEs in diabetic skin tissues can lead to a decline in the synthesis and secretion of growth factors by reparative cells, resulting in reduced levels of local growth factors at the wound site [73]. Furthermore, long-term disturbances in glucose and lipid metabolism can downregulate the transcriptional function of HIF-lα, impairing tissue cell responsiveness to hypoxia [74]. Consequently, this leads to a reduction in the activity of various growth factors downstream of HIF-lα, such as VEGF and PDGF, thereby delaying neovascularization [75]. Moreover, the glycation of growth factors and their receptors at the wound site can alter cytokine activity, diminishing the ability of growth factors to regulate wound repair.

Therefore, regarding diabetic wound healing management, it is crucial to carefully balance local inflammation and angiogenesis. An effective approach involves strengthening preventive measures and early detection, inhibiting proinflammatory effects, enhancing initial wound support, and selecting appropriate therapeutic interventions to control local inflammation and regulate neovascularization processes, ultimately facilitating optimal wound repair (Fig. 4).

#### Altered MMP/TIMPs ratio

The concept of ‘MMP/TIMP balance’ refers to the dynamic equilibrium between MMPs and TIMPs. MMPs are a group of enzymes that can degrade ECM proteins, and they play critical roles in processes such as ECM remodeling, cell migration, tissue repair, and inflammatory responses. However, their excessive activation or abnormal expression can lead to pathological tissue destruction, seen in conditions like tumor invasion and metastasis, arthritis, and atherosclerosis. To control the activity of MMPs and regulate their function, cells produce TIMPs, which are natural inhibitors of MMPs, binding to them to inhibit their activity, thus ensuring the stability and integrity of the matrix.

The MMP/TIMP balance plays a vital role in ECM remodeling and tissue repair. An increase in MMP activity facilitates cell migration and tissue repair when the ECM needs remodeling or repair. However, excessive MMP activity or insufficient TIMP expression can result in excessive ECM degradation, causing pathological conditions. Conditions like hyperglycemia, hyperlipidemia, and oxidative stress can boost the secretion of proinflammatory factors such as TNF-α and IL-1β, stimulate inflammatory cells to release MMPs, and inhibit TIMP expression. This disturbance in the MMP/TIMP balance can degrade the ECM and delay wound healing in diabetes (Fig. 5) [76].

### Timing of treatment

The timing of treatment is a significant factor influencing the wound healing process in diabetes. Efficient angiogenesis, which improves the local blood supply, is vital in treating diabetic wounds. Various therapeutic strategies can be more effective when applied at different stages of diabetic wound repair. During the early stages of wound healing, factors that promote angiogenesis may increase the proliferation and migration of endothelial cells and facilitate lumen formation. During the later stages of wound healing, factors that encourage smooth muscle cell differentiation and pericyte recruitment may be more beneficial. The use of novel materials can also promote angiogenesis and facilitate diabetic wound repair. Studies have suggested that using angiogenic factors such as VEGF and PDGF can improve wound healing in diabetes [77,78]. Cellular and gene therapy strategies have shown promise in promoting diabetic wound repair by enhancing angiogenesis [79–81]. Furthermore, certain drugs, such as angiotensin-converting enzyme inhibitors, statins, and some antioxidants, have been shown to have proangiogenic effects and may aid in diabetic wound healing [82–87] (Table 1). Although these methods have shown some success, more research is needed to determine the optimal timing and dosage for these treatments.

Nevertheless, the use of angiogenic factors alone cannot sufficiently treat diabetic wounds. The timing of angiogenesis is also crucial. The angiogenic capacity of diabetic patients is significantly reduced, which is a key factor in the difficulty of diabetic wound healing [88]. Although early angiogenesis can promote wound healing, excessive angiogenesis can lead to scar formation and tumor development. Therefore, choosing the appropriate timing of treatment can maximize the benefits of wound healing while minimizing side effects.

Researchers have also explored inducing exosome formation and reprogramming macrophage expression to regulate the balance between M1 and M2 macrophages, which are crucial in the inflammatory response. Exosomes derived from M2 macrophages have been shown to induce the complete transformation of M1 macrophages into M2 macrophages, thereby promoting wound healing through enhanced angiogenesis, re-epithelialization, and collagen deposition [89].

In conclusion, angiogenesis plays a crucial role in diabetic wound repair. Angiogenesis impairment in diabetic patients significantly contributes to delayed wound healing. Recent clinical trials have explored strategies to enhance angiogenesis [90–100] (Table 2). Therefore, promoting angiogenesis could be the key to improving diabetic wound repair. The selection of treatment strategies should consider the different stages of wound healing. Future research should focus on understanding the impact of angiogenesis on diabetic wounds.

## Conclusions

Angiogenesis is crucial for tissue regeneration in diabetic wounds. Effective intervention is essential for modulating healing and promoting tissue repair. Integrating regenerative biomaterials with immunomodulatory therapies shows promise, but clinical translation is challenging. Targeted angiogenesis therapies using extracellular vesicles are still experimental. More research is needed to improve outcomes for diabetic wound healing, focusing on the underlying mechanisms to develop effective treatments.

## Abbreviations

AGEs: Advanced glycation end products; bFGF: Basic fibroblast growth factor; ECM: Extracellular matrix; eNOS: Endothelial nitric oxide synthase; EPCs: Endothelial progenitor cells; HIFs: Hypoxia-inducible factors; IL-17RB: Interleukin-17 receptor B; MMPs: Matrix metalloproteinases; NO: Nitric oxide; PDGF: platelet-derived growth factor; PEDF: Pigment epithelium-derived factor; TGF-β: Transforming growth factor-beta; TIMP: Tissue inhibitor of metalloproteinase; TNF-α: Tumor necrosis factor-alpha; Tregs: Regulatory T cells; VEGF: Vascular endothelial growth factor.
